# Transcriptomics Insights into Spinal Cord Injury for Therapy Development

**DOI:** 10.3390/ijms27135870

**Published:** 2026-06-29

**Authors:** Daria Chudakova, Olga Astakhova, Matthew Shkap, Ekaterina Levichkina, Alesya Soboleva, Artur Biktimirov, Vladimir Baklaushev

**Affiliations:** 1 Center for Precision Genetic Technologies for Medicine, Engelhardt Institute of Molecular Biology of the Russian Academy of Sciences, 119991 Moscow, Russia; 2 Laboratory of Neuroregeneration, Federal Center for Brain and Neurotechnologies, Federal Medical and Biological Agency of Russia, 115682 Moscow, Russia; 3 Center for Cybernetic Medicine and Neuroprosthetics, Federal Center for Brain and Neurotechnologies, Federal Medical and Biological Agency of Russia, 115682 Moscow, Russia; 4 Department of Optometry and Vision Sciences, The University of Melbourne, Parkville, VIC 3010, Australia; 5 Federal Scientific and Clinical Center for Specialized Types of Medical Care and Medical Technologies, Federal Medical and Biological Agency of Russia, 115682 Moscow, Russia; 6 Department of Medical Nanobiotechnology, Medical and Biological Faculty, Pirogov Russian National Research Medical University, Ministry of Health of the Russian Federation, 117997 Moscow, Russia

**Keywords:** spinal cord injury (SCI), RNA-sequencing (RNA-seq), transcriptomics, mesenchymal stromal/stem cells (MSCs), SCI, single cell RNA-seq (scRNA-seq), single nucleus RNA-seq (snRNA-seq)

## Abstract

Traumatic spinal cord injury (SCI) is a severe medical condition, often resulting in permanent disability, with significant impacts on patients’ quality of life and burden on healthcare systems. Current therapeutic approaches for SCI are insufficient, advocating for the development of more effective treatments. As changes in transcriptome post-SCI can provide clues for novel treatment strategies and targets, substantial efforts have been made recently to characterize such transcriptional changes and their spatiotemporal features. This narrative review focuses on how transcriptomics, alone or in combination with other omics data, can contribute to understanding SCI pathobiology and the mechanisms of post-SCI regeneration and guide the development of novel SCI therapies. It covers an arsenal of tools for transcriptomics studies and provides a concise summary of findings from the latest relevant studies (predominantly from 2020 to 2025), representing the major directions in the field.

## 1. Introduction

Traumatic spinal cord injury is a debilitating neurological condition, resulting from trauma to the spinal cord (SC), with profound effects on motor, sensory, and autonomic function and high disability and mortality rates. According to the World Health Organization, in 2021, approximately 15.5 million people lived with SCI, including its predominant form, traumatic spinal cord injury (hereafter referred to as SCI) [1]. The central nervous system (CNS) of adult humans has very limited regeneration capacity, and current treatments of SCI are still insufficient and mostly focus on rehabilitation. Therefore, SCI is a “research hotspots” in translational biomedicine.

SCI is divided into complete and incomplete injuries and primary and secondary (acute, subacute, intermediate, and chronic) phases. Each phase is unique in terms of the bio-mechanical changes in the SC and surrounding tissue, resulting in shifts in their cellular composition and cell state, with metabolomic, proteomic, transcriptomic, and other changes evoked. It also has systemic consequences and a number of fundamental biological processes (immune response, sleep, etc.) are affected by it.

Several novel SCI therapies are being explored, encompassing both biotechnological and neurophysiological approaches. Biotechnological strategies include the use of bioactive molecules and pharmacological agents [2], RNA interference (RNAi) [3], neural tissue engineering, and biomaterial-based and various cell-based treatments [4], as well as recent advances in in vivo cell reprogramming [5]. Neurophysiological interventions include brain–spine interface (BSI) technologies [6], neuromodulatory techniques such as epidural electrical stimulation [7,8], photobiomodulation (PBM) [9], and others. Still, clinical approaches to the most life-threatening symptom of SCI—autonomic dysreflexia—are limited, and the lack of complete understanding of cellular and molecular events during SCI poses an obstacle to developing innovative therapies. Therefore, precise and sensitive molecular tools, such as transcriptome analysis, are needed to fully characterize molecular and cellular aspects of SCI.

The transcriptome is a “blueprint of RNA levels in the cell in a given moment”. Determining differentially expressed genes (DEGs) or broader, differentially expressed RNAs (DERs) post-SCI, including at different time-points in various parts of SC and surrounding tissues, with and without therapeutic interventions, can (i) reveal the dynamics of specific cell populations (as potential targets for therapy) at the lesion site or any other location, (ii) assess the impact of proposed therapeutic approaches, (iii) elucidate molecular and cellular mechanisms of recovery and neuroregeneration, and (iv) identify new targets and propose drug repositioning for SCI.

Several major milestones have been reached recently, namely, (1) the construction of a transcriptomic atlas of adult human SC with data from single-nucleus RNA-sequencing (snRNA-seq) and spatial transcriptomics [10], (2) determining spatiotemporal molecular changes in the adult SC after SCI with single-cell resolution [11], (3) single-cell and spatial atlases of SCI (Tabulae Paralytica) [12], and (4) a searchable database for gene transcriptomic changes in SCI with data from bulk RNA-sequencing (RNA-Seq) studies across nine species [13].

Given the space limitations and the impressive number of works on this topic, we present here an integrative and concise snapshot of the leading edge of research on the subject. We briefly outline its principal directions, illustrated by representative studies, and we highlight existing challenges and unresolved questions, including those that can be addressed with the use of available transcriptomic tools. We conclude by proposing an integrative strategy that combines all the capacities of transcriptomics with other omics approaches and emerging tools, harnessing the synergistic potential and scalable capacities of SCI-focused research consortia, which provide access to diverse datasets, standardized protocols, and shared analytical pipelines to address complex, multidimensional biological questions regarding the pathological and regenerative processes of SCI.

## 2. Transcriptomics for Spinal Cord Injury Research

### 2.1. Tools and Approaches for SCI Transcriptome Analysis

It is important to move beyond the outdated view that SCI transcriptomics merely estimates gene expression changes in bulk RNA isolated from the SC after injury, as modern tools and approaches offer far more, enabling researchers to answer questions that were previously out of reach with conventional techniques. The strategic selection of a transcriptomics study methodology, based on a thorough understanding of all available options (including their limitations and strengths), ensures optimal experimental design and maximizes discovery potential. This is why, here, we aim to present an overview of the large palette of transcriptomics tools (not necessarily applied to SCI research yet), to highlight their potential for future application in SCI.

The primary and most commonly used transcriptomic methods are bulk RNA-sequencing (RNA-seq) or microarrays and single-cell RNA-sequencing (scRNA-seq) or single-nucleus RNA-sequencing (snRNA-seq). The former analyze the transcriptome of the total RNA isolated from all cells in the tissue, and the latter determine the transcriptome of individual cells. Bulk transcriptome analysis is cheaper and easier to perform but fails to examine specific cell populations directly (including their spatial distribution), instead relying on indirect computational methods. scRNA-seq requires tissue dissociation into individual cells that biases toward viable cells, whereas fragile neurons become under-represented; also, such dissociation might be challenging in the damaged tissue post-SCI. snRNA-seq analyzes nuclei from frozen tissue, which leads to omitting cytoplasmic transcripts and provides an incomplete view of the transcriptome, with bias toward glial cells over neurons [14,15]. The wide variety of platforms for scRNA-seq and snRNA-seq, as applied to SCI transcriptomics, is comprehensively reviewed by Maihemuti et al. [16]. The more complex “molecular instrumental” can also be used. Ribosome-associated transcriptomics involves isolating and analyzing mRNAs that are attached to ribosomes and gives a snapshot of the genes that are being translated (translatome). This can be achieved via methods such as translating ribosome affinity purification (TRAP) and ribosome profiling (Ribo-seq), RiboTag approach [17]; hemagglutinin-positive ribosome IP and transcriptome profiling [18], etc. Several methods exist for the subcellular mapping of mRNA, for example, in situ hybridization [19], proximity labelling [20], Halo-seq [21], or expansion sequencing (ExSeq) [22]. For example, the spatial transcriptome can be studied using light-activated proximity labeling [23]. Further, several approaches (experimental and computational) exist for the precise mapping and quantification of post-transcriptional RNA modifications, such as methylated RNA immunoprecipitation sequencing (MeRIP-Seq), m6A-specific ultraviolet crosslinking immunoprecipitation sequencing technology (miCLIP), m6A-seq (comprehensively reviewed in [24]), ICE-seq [25] and Slic-seq [26] for A-to-I RNA editing assessment, and many others. Assessment of RNA modifications yields a clearer view of the transcriptome (and ultimately the translatome), as these modifications influence RNA stability and can shape the repertoire of splice variants with varied functions. Precision nuclear run-on sequencing (PRO-seq) [27] and global run-on sequencing (GRO-seq) [28] profile nascent RNA in cells, allowing us to study real-time transcriptional changes, which could theoretically map real-time RNA-Polymerase II dynamics in injured neurons (e.g., at regeneration-associated genes), but they have not been applied in such a context yet. Notably, abundant tRNAs and rRNAs are commonly depleted from the RNA pool at the stage of sample preparation and processing. However, they are important contributors to the post-SCI cellular response. Bulk small non-coding RNA-sequencing (sRNA-sequencing) allows expression profiling of small RNAs, such as miRNAs, piRNAs, and tRNA-derived fragments [29]. Recently, parallel single-cell small RNA (sRNA) sequencing (PSCSR-seq) has been developed for high-throughput sRNA transcription profiling of individual cells [30]. The combination of more advanced methods with more common transcriptomic approaches, or other methods of analysis, gives a deeper understanding of the molecular mechanisms of SCI. For example, patch sequencing (Patch-seq) is a technique allowing both the electrophysiological recording and transcriptomic analysis of the same neurons [31].

This can potentially permit testing neurons affected by inflammation, oxidative stress, etc., which accompany SCI, to determine the relationship between their ability to transmit signals and their transcriptomic profile to study the molecular pathways involved in either the loss or preservation of signaling. Patch capillaries also can be used for visual guidance to select particular subpopulations of neurons for sequencing, especially in vitro [32]. Thus, Patch-seq can provide transcriptomic-based insights into neuronal regenerative heterogeneity to identify drivers of neuroregeneration in the context of SCI. It could also bridge gaps in knowledge by correlating post-injury electrophysiological deficits (e.g., hyperexcitability) with transcriptomics, translatomics, or epitranscriptomic changes in neurons, hence guiding targeted therapies. Of note, transcripts within extracellular vesicles (EVs) play an important role in SCI pathogenesis, but determining the RNA cargoes of EVs derived by particular cell populations is limited to controlled in vitro settings. Further, single-cell transcriptome analysis captures RNA snapshots within individual cells but misses dynamic transfers, such as RNA shuttled from one type of cells to another via EVs [33], gap junctional channels (GJCs), or perhaps tunneling nanotubes (TNTs)—common ways of cell-to-cell communication within the central nervous system (CNS). Hence, it cannot determine with absolute certainty the cell source of the detected RNAs. Standard RNA-seq does not reliably distinguish between intact and fragmented (degraded, processed within the cell) RNA. Some pools of mRNA might be “detained”/sequestered away from ribosomes in an inactive form; therefore, the mere abundance of RNA does not accurately reflect its functional availability.

Finally, the study of the levels and spatiotemporal distribution of selected RNAs in living cells or in vivo [34] represents a potentially transformative research avenue; yet, it remains underexplored in the context of SCI, likely due to its technical challenges. Here, we acknowledge that the approaches described above do not represent a complete, exhaustive list, but they rather provide a superficial overview of the current methodological “toolkit”, serving as a necessary starting point for readers’ further exploration of the field.

**Figure 1:**
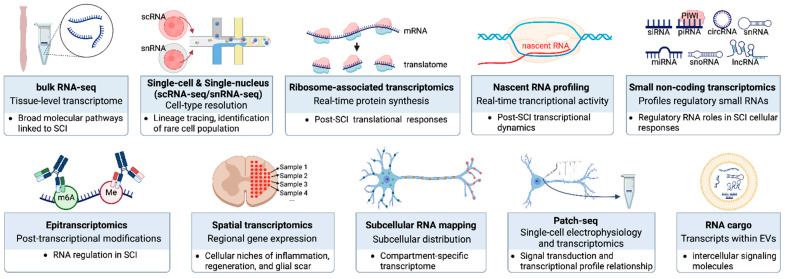
Overview of the key transcriptomic approaches applicable to SCI research. The figure summarizes the major methods used to investigate gene expression, RNA regulation, and RNA localization after SCI, spanning tissue-level, single-cell, and subcellular analyses. Created with BioRender.com. Retrieved from https://BioRender.com/jhpty7o (accessed on 8 June 2026).

We also exclude from this review various bioinformatic approaches to transcriptome analysis, while acknowledging their critical importance for the correct interpretation of the data. Collectively, these complementary technologies provide a multidimensional framework for studying pathogenesis and regeneration in SCI (Figure 1).

### 2.2. Transcriptomics Studies in Animal Models of SCI

Several animal models of SCI exist in terms of the intervention method, namely, contusion (the most representative one), transection, compression, dislocation or distraction, and neurotoxic ones [35]. Notably, the transcriptomics profiles are different depending on the type of the model [36] and also on the level/region of the injury [37]. Such models are based on regenerative vertebrates (RVs) capable of regenerating anatomically complete SCI and non-regenerative vertebrates (NRVs) [38,39]. Regenerative (R) vs. non-regenerative (NR) stages of development also exist and are taken into consideration in SCI models [40]. The ultimate goals of using the transcriptome in different models are (i) to reveal the specific molecular features of response to SCI in RV and NRV and (ii) to characterize molecular and cellular events taking place post-SCI (including their timeline) for a more targeted approach to therapy. Transcriptome studies focusing on mechanisms of neuroregeneration post-SCI in different species might reveal fundamental mechanisms crucial for cell phenotype plasticity, cell fate changes, lineage commitment, and neuroregeneration. For example, a transcriptomic study has revealed the dependence of axonal regeneration on glycosylation switch in spiny mice (*Acomys cahirinus*)—mammals capable of functional recovery after complete SC transection [41]. Many transcriptomics studies of SCI and neuroregeneration are performed in *Danio* sp. or *Xenopus* sp., non-mammalian vertebrate species distant from humans and therefore not directly translatable to the clinic. Although primate models are closest to humans and have high translational relevance, the most common models are rodent-based; among them, rats resemble human SCI better than mice [38]. Models of SCI in bipedal marsupials are valuable because their patterns of spinal control of muscle coordination are more similar to humans compared to quadrupedal species [42], and because marsupials are capable of SC regrowth after anatomically complete early-age SCI; however, only limited transcriptomics data exist for this model [43].

Rodent-to-human differences are largest in corticospinal circuitry for dexterous forelimb function, dorsal horn sensory cell types, autonomic cardiovascular control, systemic immune regulation, and the host environment encountered by human cell grafts. In primates, corticospinal projections include prominent direct cortico-motoneuronal transmission to upper limb motoneurons that supports fine hand control, whereas in rodents direct cortico-motoneuronal connections are rare and diminish with age and corticospinal excitation is largely mediated via interneuronal relays [44,45]. This anatomical and circuitry difference makes primate scRNA-seq, snR-NA-seq, and spatial transcriptomics particularly relevant for mapping cervical spinal interneuron and motor pools that underlie upper-limb outcomes and for interpreting transcriptome-derived biomarkers of “hand/arm recovery” in a human-relevant circuit context.

Primate transcriptomics is especially powerful for studying systemic immune dysfunction and for translating human cell therapies due to potential species mismatches. Primate SC offers a uniquely human-like niche for profiling graft and host states (synaptogenesis, stress and immune responses, etc.) over clinically relevant time scales [46]. Transcriptomics in such primate graft settings can therefore be used not only to describe “what cells survived” but also to quantify whether grafts reach the desired neuronal/glial transcriptional states, whether host immune programs resemble those in human SCI, and which molecular programs correlate with functional recovery. However, primate models are considerably more expensive, time-consuming, and limited by ethical restrictions; thus, there are scarcely any transcriptomic data from primate-based studies, except for a few ones [47,48], whereas an overwhelming number of transcriptomic studies of SCI have been performed using other species, including studies across a timeline after injury [11,49], in young and aged animals [50], etc. In non-human primates, studies have identified region-specific cellular/molecular responses to SCI from acute to chronic phases and suggested that a functional collagen scaffold can therapeutically remodel the degenerative microenvironment below the lesion site [47,48]; they also revealed the similarity of molecular events post-SCI in primates and rodents, but, in primates, the inflammatory response was significantly prolonged and the onset of glial scar formation was delayed, indicating different therapeutic time windows.

A major recent advance in SCI transcriptomics is the generation of single-cell atlases in injured primate SC that explicitly resolves region-dependent cellular dynamics around lesions. A single-cell study in a rhesus monkey (*Macaca mulatta*) model of SCI mapped cellular composition and gene expression programs across areas of the spinal cord proximal or distal to the injury. It revealed that the injury response was not spatially uniform and that transcriptomic signatures depended strongly on tissue sampling location and distance from the lesion [47]. This has direct methodological implications for bulk RNA-seq studies, where variable dissection strategies can create apparent discrepancies in “SCI signatures” across cohorts. Primate atlases can help address the following recurring limitation in rodent-led transcriptomic discovery: the risk that key cell subtypes and activation patterns differ between rodents and primates. These datasets can be used as (1) reference atlases for the deconvolution of bulk RNA-seq from injured spinal cord, and as (2) benchmarks for mapping candidate “rodent-derived” cell-state markers onto primate/human cell states before advancing targets.

Apart from using animal models, human SCI transcriptome datasets (for example, derived from peripheral white blood cells) can also be used to reveal potential therapeutic targets or biomarkers [51].

Rodent transcriptomic findings should therefore be interpreted as a discovery lay-er rather than as directly actionable human therapeutic evidence. SCI translation provides cautionary examples showing that biologically plausible and animal-supported mechanisms can fail to meet primary clinical endpoints. Riluzole showed favorable preclinical and early clinical signals, but the phase III RISCIS trial did not achieve its predetermined primary efficacy endpoint, although secondary and subgroup signals were reported [52]. Similarly, intrathecal anti-Nogo-A antibody therapy targeted a well-supported growth-inhibitory pathway and had extensive preclinical rationale, but a randomized phase 2b trial in acute cervical SCI did not demonstrate significant benefit on the primary upper-extremity motor endpoint across the full study population [53]. These examples are not strictly transcriptomics-driven failures, and they should not be presented as such; rather, they illustrate why transcriptomic target nomination should be coupled to cross-species validation, functional perturbation, clinically meaningful endpoints, and predefined go/no-go criteria before translation to human SCI trials.

The evidence hierarchy for SCI transcriptomic targets (a study pipeline) seems to be as follows: (i) in vitro cell-based and organoid models, which are best suited for mechanistic screening but have limited organism-level validity; (ii) rodent models, which allow causal testing, time-course sampling, genetic perturbation, and behavioral readouts; (iii) cross-model and cross-species transcriptomic comparisons, which help identify conserved injury programs; (iv) large-animal and non-human-primate models, which are particularly important when anatomical scale, corticospinal organization, autonomic regulation, immune responses, surgical delivery, or human-cell graft biology are central to the proposed therapy; and (v) human tissue, biofluid, and clinical datasets, which provide the highest translational relevance but are usually limited by heterogeneity, sparse sampling, and restricted access to spinal cord tissue.

Overall, species-specific transcriptome comparisons in the aforementioned models revealed immune response, neurogenesis, cellular proliferation, and extracellular matrix remodeling as the key targets for therapy. For example, a meta-analysis of genome-wide spinal cord gene expression datasets, including microarray and RNA-seq data (bulk RNA-seq, scRNA-seq, and snRNA-seq) at different time-points post-SCI and spanning multiple RV and NRV species (*Ambystoma mexicanum*, *Danio rerio*, *Mus musculus*, *Rattus norvegicus*, *Macaca mulatta*, *Xenopus tropicalis*, and others) was performed recently [54]. For all species, the most significantly affected pathways were cell cycle, DNA replication, neuroactive ligand–receptor interaction, metabolic pathways, apoptosis, purine metabolism, and pyrimidine metabolism. For NRV species, the significantly affected pathways were complement and coagulation cascades, neurodegeneration processes, inflammatory mediator regulation of TRP channels, and IL-17-, tumor necrosis factor-, cAMP-, and PI3K-Akt signaling pathways. Based on this analysis, cyclin-dependent kinase 1 (CDK1) reemerged as a potential target for SCI [54]. The comparison of post-SCI transcriptomes at acute and subacute phases of one RV species, axolotl, with NRV species, mice and rats, revealed the marked downregulation of Mitogen-Activated Protein Kinase (MAPK) signaling after SCI [55]. The comparison of transcriptomes in R vs. NR stages in RV species *Xenopus laevis* revealed the key role of specific ribosome biogenesis factors (in particular, rapid and transient activation of mammalian target of rapamycin complex 1, mTORC1, signaling) in the activation of neural stem progenitor cells (NSPCs); such an activation might be due to a decrease in mRNA levels of two negative regulators of mTOR, deptor and ts2 [56]. This finding reinforces the view that ribosome biogenesis is important for stem cell maintenance.

There is an ongoing debate whether progenitor cells exist in the adult SC across species commonly used in SCI studies, including humans. It has been suggested that ependymal cells in the central canal of the SC can act as quiescent neural stem/progenitor cells in adults and contribute to neuroregeneration post-SCI. Indeed, in a highly regenerative species such as axolotl, SCI evokes developmental-like programs in ependymal cells; they undergo marked acceleration of cell cycle and proliferate rapidly, ultimately driving SC regeneration [57]. However, a recent transcriptomics study utilizing snRNA-seq [58] challenged the leading role of ependymal cells as sources of stem/progenitor cells and contributors to regeneration in the adult SC of primates (NRV species). Integrative analysis of data from the rhesus macaque model of SCI and developing human spinal cord models showed that such cells respond minimally to injury, contradicting the dominant role of ependymal stem cells in the neuroregeneration of SC. Instead, indirect data suggest astrocytes are a more injury-sensitive cell population capable of acquiring an intermediate glial state (SOX9/SOX10-positive cells), where genes from both astrocytic and oligodendrocytic lineages are co-expressed simultaneously. This indicates potential transdifferentiation between astrocytes and oligodendroglia, supported by the time-dependent increase in the proportion of such intermediate cells post-SCI. Markedly, snRNA-seq in neonatal mice revealed a SC cell population exhibiting gene signatures resembling ependymal cells, astrocytes, and radial glia, highlighting their phenotypic plasticity [59]. These cells markedly increased in numbers post-injury in neonates but not adults, mirroring the regenerative potential of the neonatal mouse spinal cord, which is lost in adulthood. Overall, based on both functional and transcriptomics studies, it is reasonable to conclude that the major role of ependymal cells in SCI response is tissue homeostasis maintenance rather than neurogenesis; however, they still hold potential as SCI therapy targets [60].

Of note, an empowering, but rather underexplored approach, is the use of transgenic animals for SCI modeling [61]. This can include Cre/loxP conditional knockout animals to test cell-type-specific responses to injury; using inducible and trackable reporters for cell fate mapping; and approaches based on opto- and chemo-genetics.

It should also be noted that despite promising preclinical results, there is a risk of translation failures or challenges moving from animal to human studies in SCI therapy. This is in part because the type of injury, treatment timing, and outcome measures differ substantially between tightly controlled animal studies and heterogeneous human injuries. As aforementioned, in animal studies (predominantly rodents with thoracic contusion or transection/hemisection injuries), blockade of Nogo A—a myelin-associated inhibitor of axonal growth—was supported by extensive preclinical work showing enhanced axonal sprouting and regeneration and functional recovery. Notably, in macaque models of cervical SCI, intrathecal administration of anti-Nogo A antibodies was reported to accelerate and improve recovery of skilled forelimb function (manual dexterity) as well, and to enhance corticospinal tract sprouting below the lesion [62,63]. These non-human primate results, together with extensive rodent data, are summarized in translational reviews noting that anti-Nogo A approaches showed efficacy and were evaluated for safety in both rodents and non-human primates prior to progression into clinical testing [64]. Translation to humans was tested in a randomized, double-blind, multicenter, placebo-controlled phase 2b trial using intrathecal anti-Nogo A antibodies in acute cervical traumatic SCI, i.e., a markedly different context than in many rodent studies which utilized different spinal level, larger and more heterogeneous lesion patterns, and clinically constrained treatment windows. In the aforementioned human study, the treatment was safe but did not demonstrate a significant benefit on the primary efficacy outcome, highlighting the challenge of extrapolating from relatively homogeneous rodent injury models and behavioral outcomes to complex, variable cervical SCI in humans [53]). Another example is Riluzole (blockator of glutamatergic neurotransmission), which appeared to be neuroprotective in multiple preclinical works, reducing excitotoxicity and neuronal firing burden in animal SCI models with precisely controlled injury severity and early drug delivery. The translational test was the multicenter, randomized, placebo-controlled, double-blinded RISCIS trial in acute cervical SCI, where real-world constraints (time to treatment, care, and injury heterogeneity) are far more variable than in experimental models. The trial did not meet its prespecified primary endpoint, exemplifying how a mechanistically plausible intervention can produce weaker or more variable outputs when moved from standardized animal studies to human trials [52]. These examples highlight a common translational mismatch as follows: beyond inherent species differences, animal studies often under-represent the subject heterogeneity, comorbidities, and complication profiles that determine human outcomes [65]. Nevertheless, when these limitations are duly considered, animal studies (including transcriptomics) remain instrumental in SCI research.

### 2.3. Subtranscriptome of Cellular Compartments, Translation of Subsets of mRNA (Translatome)

Changes in the subcellular localization/abundance of particular RNAs (subtranscriptome) have a significant impact on cell functions. This is especially true for neurons—highly polarized cells with long axons and free ribosomes in distal parts of the cell, enabling local and on-demand protein biosynthesis using compartmentalized “dormant” mRNAs [66]. Local regulation of the subtranscriptome in neurons is achieved through targeted transport of mRNA into axons within transport granules, mediated by RNA-binding proteins (RBPs), their storage in “dormant” granules, and subsequent rapid activation of translation in response to injury signals or trophic stimulation [67]. This enables the axon to function as a semi-autonomous compartment capable of altering its own proteome without awaiting protein transport from the cell soma. One of the key mechanisms for controlling the availability of axonal mRNAs is their temporary storage in aforementioned granules (also known as “stress granules”). After axotomy of mammalian neurons, injury-activated degradation of stress granule-like structures occurs, along with the initiation of local translation of previously stored transcripts. The protein G3BP1 plays a central role in this process. Its aggregation correlates with translation suppression, whereas granule disassembly is accompanied by its phosphorylation, axon growth, and regeneration [68]. This indicates the possible role of the G3BP1 and axonal subtranscriptome in axon recovery post-SCI. Therapeutic targets for SCI can also be translated at specific subcellular sites, which might be crucial for their functionality. For example, microtubule severing enzyme (MSE) Fidgetin Like 2 (Fl2) inhibits axonal growth and becomes elevated post-SCI [69]; several lines of evidence position it as a potential target for nerve regeneration ([70] and others, discussed in detail further in the text). Notably, it has been demonstrated that Fl2 mRNA is mainly localized at the leading edge of cells and is translated locally in response to cell injury [71].

Bulk RNA-seq of RNA from the axonal subcellular fraction in a rat SCI model identified DEGs linked primarily to axonogenesis [72]. Unexpectedly, circRNAs, including circRims2, constituted a significant portion of differentially expressed transcripts. Supposedly, they may regulate the local translation of key regenerative proteins such as GAP-43, PTEN, and CREB1. The knockdown of ADAR1, which limits circRNA formation, enhanced axonal growth, indicating the critical regulatory role of circRNAs in local protein synthesis control and axon recovery.

Analysis of scRNA-seq data from micro-aspirated axoplasms from growing, static, and retracting axon tips of transection SCI in larval lampreys (*Petromyzon marinus*) identified genes related to axonal regeneration, such as *map3k2* (involved in the MAPK pathway) and *csnk1e* (involved in circadian rhythm signaling); high mRNA diversity and unique state-specific mRNAs in growing axonal tips reinforced the view that local protein synthesis drives axon regeneration [73].

Notably, it has been demonstrated that axonal regeneration after CNS trauma in mice post optic nerve crush injury depends on the translation (rather than transcription) of a specific (rather than the total) subset of mRNAs, regulated by ribosome-interacting proteins [74]. It has been suggested that the repair of neuronal circuits can be achieved via increasing the translation of these subsets of mRNA. Whether the same is true in the case of axonal regeneration post-SCI is a burning question which can be addressed using a combined transcriptomics/translatomics approach (we also note that retinal ganglion cells receive constant stimulation from the other retinal cells as long as the retina is intact). Schaeffer J et al. also raised the question whether such selective translation differs de-pending on localization in cell stroma/axon shaft/axon growth cones.

The elements determining RNA subcellular localization are not fully characterized for the majority of RNAs yet; the most well-studied elements include the so-called “zipcode sequences” in 3′ untranslated regions (3′-UTRs) of mRNA [75]. Notably, axonal localization elements may also reside in the 5′UTR; for example, such elements were identified in the short 5′UTR of mTOR, alongside that known in 3′UTR [76]. The mTOR pathway (comprising two complexes, mTORC1 and mTORC2) is one of the key regulators of neuronal regeneration, proliferation, and differentiation of NSCs [77]. It has been demonstrated in mice that the mRNA of mTOR is transported to axons allowing its rapid local translation following upregulation in response to axon injury [78]. This, in turn, regulates the translation of several molecules involved in retrograde long-range injury signaling (e.g., importin beta 1). Not surprisingly, therapies utilizing the inhibition of PTEN—negative regulator of mTOR signaling—show considerable promise for SCI therapies [79]. Both mTOR inhibition or the prevention of its localized translation (via deletion of the 3′-UTR containing the axonal localization signal) reduced neuronal survival after the injury [78], confirming its crucial role in neuroregeneration. In light of this, when developing mRNA-based therapies for SCI, the importance of “zipcode sequences” in the 3′-UTR should be taken into account.

RNA stability can be regulated by RNA-binding proteins (RBPs), they also govern mRNA trafficking to subcellular compartments and localized protein synthesis. RNA-seq analysis of mice with SCI or sham group revealed several RBPs associated with SCI (four major—*Nkrf, Marcks, NDRG4*, and *Ryr2*). Several genes with alternative splicing evoked by SCI have also been found, for example, *App*, *Chl*, *Cdc42*, *Kif 2A*, *Nptn,* and *Rtn4*. Processes in which RBP-related alternatively spliced genes are involved post-SCI are synaptic organization, axon formation, synaptic guidance, and GTPase. It has been suggested that these key RBPs might become targets for SCI therapy [80].

### 2.4. Non-Coding Transcriptome

Most of the human genome is non-coding, but at least 70% of the genome is transcribed, generating a diverse repertoire of RNAs that collectively shape cellular phenotype and function. The non-coding transcriptome comprises multiple classes of RNAs, including long non-coding RNAs (lncRNAs), circular RNAs (circRNAs), microRNAs (miRNAs), and other small RNAs, namely, small nucleolar RNAs (snoRNAs), Piwi-interacting RNAs (piRNAs), small nuclear RNAs (snRNAs), etc., which control mRNA stability, maturation, translation, transposon activity, and other molecular processes, thereby determining how the protein-coding transcript pool is utilized and ultimately shaping cell fate [81]. Thus, the whole coding and non-coding transcriptome—including the ribosomal RNA (rRNA) which is commonly depleted from the pool of RNA subjected to transcriptome analysis—should be analyzed in the context of SCI. Generally, the role of non-coding RNAs (ncRNAs) in SCI and neuroregeneration is well-reconfigured [82,83,84]. There are several recent comprehensive reviews on miRNA and lncRNAs in SCI [85,86]. Several targets based on miRNA transcriptome analysis have been proposed for SCI therapy, for example, upregulation of miR-182 [87] or modulation of miR-21 [88], subject to further preclinical and clinical validation. The research progress on miRNAs in SCI is impressive (summarized in [89]), and many targets identified by transcriptomics studies have been functionally validated in animal models of SCI. To name but a few, knockdown of miRNA-324-5p in rats with SCI attenuated neuronal loss and locomotor deficits, preserving expression of neurotrophic factors BDNF and GDNF [90]. In a rat SCI model, overexpression of miRNA-124 promoted functional recovery by reducing lesion size and suppressing neuronal apoptosis [91]. Further, circRNAs may “sponge” miRNAs and therefore act as competing endogenous RNAs. Some circRNAs play a role in the regulation of axonal growth post-SCI, therefore manipulating their levels might become a therapeutic strategy [92]. For example, circRNA_01477 was found markedly elevated after the SCI [93] and its knockdown significantly increased the length of axons [94]. CircRNA-2960 is a validated causative factor in the inflammatory and apoptotic processes occurring at lesion sites in a rat model of SCI; disruption of its expression promoted recovery of tissues impacted by secondary SCI damage [95]. Notably, its target is the aforementioned miRNA-124. Indeed, in the context of SCI, the dynamics of different types of non-coding RNAs must be examined as a system of interconnected events, not as isolated changes.

Of interest is also the role of less studied regulatory ncRNAs, such as transfer RNA-derived small RNAs (tsRNAs), which act similarly to miRNAs as gene expression regulators. For example, some tsRNAs could target mRNA encoding BDNF, which is crucial for neuronal survival, axonal growth, and synaptic plasticity [96], suggesting that ncRNA-mediated modulation of BDNF may represent a therapeutic avenue. rRNAs are indispensable for ribosome biogenesis and its catalytic capacity, processes increasingly recognized as involved in injury response and tissue regeneration. Notably, ribosomal biogenesis transiently increases at the injury epicenter after SCI in mice and rats, yet pharmacological inhibition of rRNA transcription does not markedly alter locomotor recovery [97]. It was speculated by the authors of this study that the production of specialized ribosomes—a subset of ribosomes which differ in composition and preferentially translate a specific subset of mRNAs—might be triggered by SCI to optimize the translation of transcripts involved in injury responses. Indeed, the expression of genes encoding ribosomal proteins is significantly suppressed during neuronal maturation but partially reactivated after injury, possibly to form a “pro-regenerative” translational machinery. Experimental overexpression of individual genes encoding ribosomal proteins involved in CNS development process, particularly *Rpl7* and *Rpl7A*, enhanced axon regeneration in vivo, indicating the existence of specialized ribosomal proteins predominantly responsible for translating mRNAs associated with axonal growth programs [98]. Finally, it has been shown recently that a specific subfamily of transposable elements—a subset of polyadenylated B2-Short-interspersed nuclear elements RNAs (hereafter referred to as growth-inducing B2-SINEs, GI-SINEs)—are induced by nerve injury and interact with ribosomal proteins and nucleolin, hence regulating translation in neuronal cytoplasm. Overexpression of exogenous GI-SINEs enhanced axon out-growth in corticospinal neurons [99].

### 2.5. Post-Translational Modifications of Transcriptome (Epitranscriptomics)

Epitranscriptomics (also known as editomics) are co- and post-transcriptional chemical modifications of both mRNA and non-coding RNAs, such as the most studied N6-methyladenosine (m6A), N6,2′-O-dimethyladenosine (m6Am), N1-methyladenosine (m1A), 5-methylcytosine (m5C), Pseudouridine (Ψ), N4-acetylcytosine (ac4C), N7-methylguanosine (m7G) modifications, and adenosine-to-inosine (A-to-I) editing, that control critical steps of RNA metabolism, such as splicing, export, localization, stability, degradation, translation, tertiary structure, etc. [100]. Such modifications of RNA are also involved in the pathogenesis of CNS injuries, including SCI [101,102]. For example, Yu et al. performed MeRIP-Seq and created a map of m6A in rat SC in the subacute phase of SCI [103]. They found that the expression of the *Ngf* gene, encoding the nerve growth factor, increases post-SCI, and *Ngf* mRNA becomes hyper-m6A-methylated. The authors raise the question whether drugs affecting m6A should be tested in the context of SCI. This approach appears feasible, given the diverse therapeutic strategies that can be used to target the epitranscriptomic machinery [104]. The relationship between m6A and SCI is comprehensively discussed in a recent review by Liu et al. [105], highlighting possible targets such as METTL3/14 “writers”, FTO/ALKBH5 “erasers”, and YTHDF “readers” of m6A. How they change after SCI remains to be further investigated. Another question is whether other types of RNA undergo other significant (and druggable) modifications associated with SCI and affecting their functions.

Here, we emphasize that it is important to establish whether epitranscriptomic changes after SCI are merely a consequence of trauma or also active regulators of key processes. Evidence suggests the latter is possible, pointing to epitranscriptomic changes and their associated enzymes as potential therapeutic targets. For example, m5C modifications are known to be affected by SCI [106]. A recent comprehensive study, combining gene expression analysis, RNA m5C dot blot, and m5C RNA immunoprecipitation (RIP), demonstrated that functional recovery after SCI can be promoted by the neuroprotective compound tetramethylpyrazine (TMP). This occurs through reducing the m5C modification of *XBP1* mRNA via the NSUN2-XBP1 pathway [106]. The aforementioned epitranscriptomic study further supports the previously suggested clinical value of TMP for SCI, validates its mechanism of action as an epitranscriptome modulator, and once again highlights the driving role of mRNA m5C modifications in SCI pathogenesis. Notably, m5C modifications occurring in tRNAs, rRNAs, and mRNAs also appear to be involved in SCI [107] and yet remain to be thoroughly studied. Another recent study reported decreased levels of METTL16 protein in the SC after SCI and showed that METTL16 inhibits neuronal apoptosis through modulation of m6A modifications on *MCEMP1* mRNA [108]. Yet another recent epitranscriptomics study provides further evidence of the causal role of RNA modifications in SCI trauma and regeneration, identifying a potential druggable target. Specifically, m6A methylation was shown to regulate corticospinal tract (CST) regeneration after SCI. The mechanism involved the METTL14-mediated regulation of *Trib2* expression through m6A RNA modification, which subsequently activates the MAPK pathway. Administration of syringin, a METTL14 stabilizer, enhanced CST regeneration and promoted neurological recovery [109]. Hence, epitranscriptomic changes in SCI emerge as active drivers of both pathological and regenerative processes. This dual role positions them as a valuable source of potential therapeutic targets, warranting further research in this field.

### 2.6. Extracellular Vesicle Transcriptomes in SCI

EVs, including exosomes and microvesicles, membrane-bound nanovesicles ~50–1000 nm in diameter (50–250 for exosomes and up to 1000 nm for microvesicles), hold great potential for SCI therapy when they are derived from cells with neuroprotective properties, such as mesenchymal stromal/stem cells (MSCs) or others [110,111]. Such EVs are proposed as therapy for SCI, therefore transcriptomic analysis of their molecular load is instrumental in studies optimizing such therapy. At the same time, as components of a cell-to-cell communication network, they can contribute to SCI pathogenesis/modulate injury response through the molecular contents of their cargoes. A recent comprehensive review focuses on EV-based therapies for SCI in detail [112]. Here, we provide several examples of using transcriptome analysis of EV to guide SCI therapies. For example, it has been found that bioactive cargo of MSC-derived EVs differs depending on tissue source and culture condition [113], which in turn might affect their therapeutic efficiency. In a mouse model of SCI, miRNA-sequencing of contents of EVs secreted by activated microglial cells revealed their enrichment with particular miRNAs, for example, miR-152-3p, compared to sham control. Such EVs enter the hippocampus and their regulatory cargoes inhibit WNT10b signaling [114]. This leads to a decrease in the proliferation and differentiation of NSCs, disrupting hippocampal neurogenesis and cognitive function post-SCI. Further, scRNA-seq of CD271+CD56+ bone-marrow-derived subpopulation of MSCs identified a repertoire of miRNAs enriched in exosomes, such as miR-431-3p, targeting pathways that inhibit regeneration (e.g., Repulsive Guidance Molecule BMP Co-Receptor A signaling). Prolonged release of these exosomes promoted axon regeneration and functional recovery in the SCI model [115]. In addition, different modifications of EVs can enable targeted delivery of their contents, hence enhancing regenerative effects. For example, scRNA-seq revealed that the receptor of Angiopep-2 peptide (Ang2) is elevated post-SCI. Engineered EVs modified with Ang2 demonstrated specific delivery of RNA cargo across the blood–spinal cord barrier, enhancing functional repair in a SCI model [116]. In another study, EVs were modified with dual ligands—Angiopep-2 and Arg-Gly-Asp—pretreated with curcumin to enhance anti-inflammatory and neuroregenerative properties. SnRNA-seq showed that such EVs reprogrammed pro-inflammatory microglia toward reparative phenotypes, suppressed neuroinflammation, promoted axon regeneration through phagocytosis of myelin debris, and supported blood-spinal cord barrier repair post-SCI in a rodent model [117]. Taken together, these studies demonstrate that the EV transcriptome is functionally active and capable of modulating inflammation, neurogenesis, and axon regeneration after SCI. The systematic transcriptomic profiling and engineering of EV RNA cargo therefore represent a rational strategy both for identifying disease-relevant biomarkers and for designing EV-based therapeutics for SCI.

### 2.7. Transcriptome for Lineage Tracing and Cell Fate Assessment

Taking transcriptomic “snapshots” at several stages of a particular process allows for mapping cell fate trajectories, including in case of cell reprogramming (transdifferentiation), differentiation and dedifferentiation. In a mouse model of SCI, scRNA-seq analysis characterized a subtype of astrocytes (differentially expressing *Gap43*, *Vim*, *Aldoc*, and *Mt1*) with neuron-like transcriptional signatures [118]. It was suggested that these astrocytes are capable of transitioning into neurons during the SCI. Based on transcriptome analysis, the authors of the study proposed that *genes Gap43*, *Vim*, *Aldoc*, *Mt1*, *Atp1b2*, and *Gpr37l1* are “hub” genes orchestrating such transdifferentiation, and that glycolytic pathways play a role in astrocyte-to-neuron (AtN) reprogramming taking place in situ. Further, astrocyte-to-induced neuron (iN) conversion (AtiN) in situ via ectopic expression (EE) of transcriptional factor NeuroD1 is a promising approach to SCI therapy [5,119]; its “driving” molecular mechanisms have been previously characterized by transcriptomic analyses [120]. The possibility of such conversion and origin of iNs generated by NeuroD1-mediated conversion are highly debated. Using spatiotemporal lineage mapping and snRNA-seq transcriptomics in an inducible NeuroD1 knock-in mouse model, it was found that such conversion can be evoked in distinct reactive astrocytes in the lesion core during early but not late phases of dorsal hemisection SCI [121] (thus, determining therapeutic time window). Contrary to previous findings, such iN did not have the neuroelectrical properties (NEPs) of mature neurons at 17 days after conversion, which questions their functional integration into neural circuits; however, NEPs at later time-points have not been assessed yet in this model. Puls et al. previously reported that, in contusion SCI in mice, such conversion can occur with a long delay post-injury. Chen et al. confirms the astrocytic origin of iN cells, but it emphasizes the need for optimization of the NeuroD1-based protocols for cell fate conversion in vivo in the context of SCI. Further studies determining if such conversion leads to functional improvement post-SCI in different models are also needed, to assess the potential therapeutic benefits of such an approach. This work also demonstrates the particular value of using lineage-traceable transgenic animals for SCI study.

The integration of snRNA-seq data from *Macaca mulatta* SCI models with human SC developmental transcriptomes revealed that ependymal cells fail to revert to a progenitor-like developmental state post-SCI, and hence they do not contribute to tissue regeneration post-SCI [58]. Subsequent lineage tracing demonstrated that, after SCI, some astrocytes transdifferentiate into mature oligodendrocytes and revealed a distinct adult glial population within SC co-expressing astrocyte and oligodendrocyte markers. Notably, functional scaffolds modulating the injury microenvironment enhanced the astrocyte-to-oligodendrocyte conversion rate [58]. As for the role of ependymal cells in NSCs formation and tissue regeneration in SCI, thoroughly discussed in one of the previous sub-chapters, another scRNA-seq study in a mouse model of SCI confirmed that ependymal cells within a central channel (cc) of SC indeed are not capable of reversing to a pro-regeneration state, but their Nestin-positive subgroup located outside the cc is activated after SCI and has NSCs properties (capability of neurosphere formation, etc.) [122].

### 2.8. Transcriptome and Cell-Based SCI Therapy

Cell-based therapy holds promise for SCI, as it has been demonstrated for several types of cells, such as neural progenitor cells (NPCs), MSCs, and others in various animal models including primates [123,124], and in clinical studies, including 26 finalized clinical trials of MSC-based SCI therapy [125]. Primate models revealed that human pluripotent stem cell-derived neuronal grafts can survive, mature, and integrate in injured SC. GABAergic neurons, which are commonly lost after SCI, survived for more than half a year, matured, formed synapses, and showed functional activity in a rhesus macaque SCI model [58]. A recent study utilized human embryonic stem cells (H9-scNSCs) post-SCI and reported significant improvement of the forelimb functions [126]. Only a few NPCs are present in adult SC of NR species. scRNA-seq revealed marked upregulation of p21 in response to SCI in rats; bulk RNA-seq analysis demonstrated that p21 RNAi knockdown in NPCs/NSCs induced their proliferation, and RNAi of p21 in vivo in T9 rat SCI led to improved outcomes in functional recovery [127]. Thus, p21 might be a promising target for SCI therapy in the context of cell-based therapy. In a study by Liu et al., neuroactive network tissue (NNT) was created by immobilizing dual recombinant growth factors onto an oriented electrospun nanofiber scaffold incorporating NSCs, and scRNA-seq showed that NNT reduces immune cell activation while promoting the survival of neurons and oligodendrocytes [128].

Transcriptome analysis confirmed that human NPCs transplanted to the SCI lesion acquire transcriptional profiles of regional SC neurons or oligodendrocytes, thus confirming their potential clinical value [129].

Another study, partially based on analysis of the transcriptome, demonstrated that the CSPGs/LAR/PTPσ axis suppresses the neuronal differentiation of human NPCs transplanted to rats post-SCI; thus, its pharmacological inhibition might promote NPCs maturation, synaptic functions, etc., after SCI [130] (notably, a PTPσ modulator NVG-291 is currently undergoing clinical trials for SCI).

As for the MSC-based therapy, transcriptomic data revealed that MSCs transplanted to the lesion site in acute SCI in mice survived for at least 7 days after transplantation [131], which further defines their therapeutic time window. In particular, RNA-seq of transplanted MSCs recovered from the injured SC demonstrated that these cells do not remain in a static “manufactured/baseline” state in vivo. Instead, MSCs downregulated cell-cycle programs and upregulated immune response, endocytosis, and phagocytosis-related transcriptional signatures, effectively adopting immune cell-like characteristics within the lesion environment [131]. This finding supports a short, early therapeutic window where MSCs may act primarily through immunomodulation rather than long-term engraftment and suggests that improving MSC efficacy may require either repeated dosing, preconditioning, or co-therapies that mitigate or block this phenotypic change in transplanted MSCs in the hostile post-SCI niche.

Transcriptomic analysis also further elucidated MOA of MSC-based SCI therapy [132]. It demonstrated that, in rats with subacute thoracic SCI, transplantation of human umbilical cord-derived MSC (hUC-MSCs) leads to suppression of lactylation-associated gene expression signatures. This finding defines lactylation-related DEGs as potential mechanistic nodes and candidate biomarkers of therapy response [132]. In parallel, mechanistic evidence from a high-impact study shows that metabolic reprogramming through histone lactylation in microglia and macrophages can recruit CD8+ T lymphocytes and aggravate SCI, reinforcing lactylation as a plausible targetable axis at the intersection of inflammation and tissue damage [133]. These studies motivate translational strategies that combine MSC delivery with therapies aimed at immune–metabolic checkpoints to prolong or amplify beneficial immunomodulation.

For NPC grafts, transcriptomics has been particularly valuable for identifying extrinsic signals in the injured SC that suppress neuronal differentiation and synaptic integration. It was demonstrated that the chondroitin sulfate proteoglycan (CSPG) environment signals through leukocyte common antigen-related (LAR) and protein tyrosine phosphatase sigma (PTPσ) receptors to inhibit neuronal replacement by human NPC grafts. Pharmacological co-blockade of LAR and PTPσ activated broad transcriptional programs supporting neuronal differentiation and synaptogenesis, including Wnt/β-catenin pathway engagement, and improved functional recovery after SCI [130]. That provides a concrete “two-component” translational strategy as follows: combine cell transplantation with targeted modulation of the inhibitory extracellular matrix–receptor axis to promote graft maturation and circuit integration, and use graft transcriptomics as a pharmacodynamic readout to confirm that maturation programs are engaged in vivo.

Finally, transcriptomic studies in the context of cell therapy may potentially identify transcriptomic signatures of transplanted cells predictive of therapeutic efficacy and help validating and optimizing preconditioning strategies (e.g., cytokine priming). For example, such approach revealed that pro-inflammatory cytokine priming of MSCs in vitro reduces the donor-related heterogeneity of immunomodulatory gene expression and enhances the immunomodulatory capacity of MSCs, and such response is not transient [134].

### 2.9. Transcriptome Analysis to Study SCI-Associated Systemic Syndromes

#### 2.9.1. SCI-Associated Immunodeficiency and Circadian Rhythm Dysregulation

SCI causes severe disruption of the sympathetic pathways of the autonomic nervous system, which, among the other systems, innervates immune organs. In SCI disruption of supraspinal inputs, especially at higher thoracic or cervical levels, interrupts descending sympathetic signals. This leads to abnormal noradrenergic and corticosteroid signaling to immune tissues and direct immunosuppression of T-cell and B-cell function by glucocorticoids [135]. As a result, SCI causes secondary SCI-induced immunodeficiency syndrome (SCI-IDS), increasing susceptibility to infections. Moreover, SCI is accompanied by circadian rhythm dysregulation caused by impaired production of melatonin [136]. Neuroinflammation also leads to disrupted circadian rhythms, creating a vicious cycle enhancing inflammatory damage [137]. Sleep disruption by SCI also results from the absence of sleep-affecting signals normally ascending by the SC [138]. Bulk-RNA-seq of several tissues in the T3 and T10 transection models of SCI in rats revealed that the degree of peripheral immunosuppression is related to the level of lesion, affects circadian rhythms, and that T3 injury impairs both splenic immune function and adrenal-mediated adaptive immune response [139]. Circadian clock genes like *Bmal1* and *Clock* drive the expression of a significant part of the transcriptome [140], including the so-called “inflammatory genes”, and SCI-IDS is further aggravated by abnormalities in circadian rhythm. Integrating chronobiology into study design—standardizing collection time, recording sleep–wake schedules of model animals, and considering light exposure—can improve the accuracy and comparability of molecular findings. This consideration is particularly relevant for transcriptomics [141,142,143]. A rat-based study showed that uninjured SC expresses rhythmic clock genes (*Per2*, *Cry1*, *Bmal1*, and *Reverbα*), but SCI reduces expression and abolishes diurnal variation at the lesion epicenter and also reduces clock-gene expression in distal lumbar SC. Thus, transcriptomic data provide evidence that SCI can reprogram local and remote spinal clocks, supporting the mechanistic premise that circadian dysregulation is a part of biology of secondary injury [144]. Therefore, sampling time is a “pre-analytical” variable for transcriptome studies and should be documented and, where possible, standardized, so that reported molecular signatures reflect disease biology rather than sampling time-related artifacts [141]. Further, restoring disrupted circadian clock mechanisms might become a therapy for secondary damage post-SCI [145].

#### 2.9.2. SCI-Associated Spasticity

Spasticity is a velocity-dependent increase in muscle tone with exaggerated tendon reflexes resulting from upper motor neuron lesions, one of the most common secondary complications after SCI. In a complete transection SCI model at sacral level S2 associated with delayed-onset tail spasms, RNA-seq revealed marked upregulation of the myeloid-lineage transcription factor PU.1/Spi1 and its downstream network. That is consistent with injury-driven shifts in immune and microglial composition and activation [146]. The most prominent were widespread changes in RNA processing: more than 1000 differential intron retention events were detected. Retained introns can introduce premature stop codons, triggering nonsense-mediated mRNA decay, nuclear retention of the transcript, or the production of alternative protein isoforms if translated. Hart et al. also highlighted a critical design issue for transcriptomic interpretation in spasticity research: even in naive SC, rostral vs. caudal segments differed substantially in both gene expression and intron retention patterns. Thus, segment selection relative to lesion can confound “spasticity signatures” unless explicitly controlled.

Overall, SCI-associated systemic syndromes remain substantially understudied by transcriptomic methods, which creates an opportunity for future transcriptomic research: longitudinal blood, immune-cell, skin, muscle, and, where feasible, organ-specific sampling and subsequent transcriptomics (potentially combined with multiomics) analysis could define molecular endotypes of systemic SCI complications and identify biomarkers of risk and treatment response. Such studies should integrate transcriptomics with lesion level, autonomic completeness, medication exposure, infection status, bowel/bladder programs, sleep and circadian timing, rehabilitation intensity, patient-reported outcomes, etc., so that molecular signatures are linked to clinical syndromes rather than to SCI diagnosis alone.

### 2.10. Identification of Therapeutic Targets and Therapy Mechanisms of Action

There are several studies utilizing bioinformatic analysis of transcriptomic data to find novel targets or therapeutic agents in SCI. RNA-seq at different time-points from acute to chronic SCI in rats followed by gene–drug network analysis of transcriptome data suggested several bioactive molecule candidates for the SCI treatment, including those already outlined by clinical neuropharmacological studies, such as dehydroepiandrosterone and Amiloride [49]. As aforementioned, based on results of a comparative cross-species transcriptional analysis the MAPK signaling was proposed as a potential therapeutic target for SCI [55]. An integrated bioinformatic analysis of transcriptome also pointed to the MAPK pathway as involved in SCI pathogenesis [147]. The therapeutic potential of targeting MAPK signaling in SCI was further explored in many works in different models [148].

An algorithm for the discovery of SCI therapeutic targets, based on analysis of transcriptome corresponding to dorsal root ganglia axon regeneration after conditioning lesion, identified telmisartan as a compound promoting axonal regeneration [149]. Analysis of bulk RNA-seq and scRNA-seq data in subacute SCI in mice identified immune hub genes and suggested that low-dose decitabine may promote SC regeneration [150]. RNA-seq in a rat contusion SCI model showed significant FL2 gene expression upregulation at the injury epicenter one day post-injury. Single delivery of siRNA-FL2 nanoparticles improved motor function moderately, reduced Iba1+ microglia, and increased CC1+ oligodendrocytes at the lesion site 28 days post-SCI. Transcriptomics analysis indicated that FL2 gene knockdown modulates chemokine signaling (*Cxcl11*, *Cxcl10*, *Ccl20*, and *Cxcl1*), pro-/anti-inflammatory immunity (*Il6*, *Il11*, *Il1b*, *Cd8a*, *Ciita*, and *Rt1-Da*), and leads to elevated expression of neuronal receptor genes (*Drd1*, *Chrnb3*, *Gpr156*, *Htr6*, and *Gabrr2*). These findings position FL2 as a negative regulator of regeneration, proposing its targeted knockdown as a therapeutic strategy for SCI [151]. The neuroprotective role of nicotinamide mononucleotide (NMN) was investigated in a mice model of contusion SCI [152]. Systemic administration of NMN restored NAD+ levels, promoted improvement in motor functions, and reduced pain syndrome after injury. Transcriptomic analysis showed that NMN suppresses inflammatory processes by regulating IL-17, TNF, toll-like, and NOD-like receptor pathways, while also decreasing the expression of pro-inflammatory cytokines and chemokines, including IL-1β, TNF-α, and Cxcl10. Thus, compensating NAD+ deficiency with NMN represents a promising, focused on metabolic processes, strategy for SCI therapy.

Translational profiling of the layer V corticospinal tract neurons in mice revealed that injured adult neurons post-SCI revert to an “embryonic transcriptional growth state.” This regenerative transcriptome profile (RTP) either diminishes after two weeks or—if mice receive neural progenitor cell (NPC) grafts—remains sustained [153]. Subsequent in-silico analysis of RTP identified several compounds evoking similar transcriptional changes, namely, quinostatin (inhibitor of PI3 kinase), thiorphan (neutral endopeptidase inhibitor), triflusal (inhibitor of nuclear factor κB, phosphodiesterase, and COX1), and milrinone (phosphodiesterase inhibitor). Follow-up tests demonstrated that thiorphan reprograms neurons to promote functional recovery post-SCI, thereby positioning it as a candidate for the treatment of SCI. It also enhanced motor function recovery when combined with NSCs transplanted to the injury site of severe cervical SCI [154]. Notably, transcriptional datasets of particular neuronal subpopulation indispensable for voluntary movements post-SCI were used for analysis, rather than datasets obtained from the “bulk” neuronal/glial population. Not only neuronal cells but also a diverse array of other types of cells at the injury site and surrounding tissue contribute to SCI pathobiology. Transcriptomic study revealed that Tregs regulate microglial synaptic phagocytosis function post-SCI through modulation of CD74 expression via the release of osteopontin (OPN). Therefore, targeting the OPN-CD74 signaling might be used as a potential SCI therapy [155].

An integrated analysis of scRNA-seq and snRNA-seq datasets from several independent studies was applied to investigate the mechanisms of microglia and astrocyte activation after SCI [156]. It was found that the CD44 receptor in microglia plays a key role in activating a pro-inflammatory microglia subpopulation through interaction with its ligand OPN, which is accompanied by activation of NF-κB-dependent pathways and increased expression of IL-6. A key astrocyte subpopulation emerging on day 3 post-injury, which becomes dominant in the injury epicenter and is characterized by high expression of nucleolin (NCL) and proliferative capacity, was identified. It was shown that the pleiotrophin signaling pathway enhances NCL expression and thereby stimulates gliogenesis. Notably, it has been shown previously that inhibition of NCL by the aptamer GroA (AS1411) can attenuate reactivity of astrocytes in SCI [157]. Overall, this transcriptomics-based work identifies CD44 and pleiotrophin/NCL signaling as key regulators of inflammation and reactive gliosis, proposing them as promising therapeutic targets for improving SC repair after SCI.

The pleiotrophin/NCL axis probably needs to be validated in a temporally and spatially resolved manner before being advanced as a therapeutic target. Indeed, the NCL-high astrocyte population identified after SCI may contribute to reactive gliogenesis, and PTN-NCL signaling has been proposed to promote astrocyte proliferation after injury [156]. However, it is well-known that astrocyte responses are not uniformly detrimental: reactive astrocytes and astrocytic scar borders can restrict inflammatory and fibrotic spread, protect spared tissue, and, under defined growth-promoting conditions, support rather than prevent axon regeneration. Conversely, excessive or persistent gliosis and scar-associated extracellular-matrix remodeling may still limit plasticity and axonal growth, indicating that astrocyte-targeted interventions require careful timing and lesion-compartment specificity [158]. Therefore, targeting NCL or pleiotrophin signaling should be tested with attention to treatment timing, lesion core versus border effects, and astrocyte-subtype specificity. Future studies should determine whether modulation of this pathway suppresses maladaptive astrocyte proliferation without impairing barrier formation, immune containment, tissue preservation, or interactions with regenerating axons.

Analysis of transcriptomic data from the mouse SC after contusion SCI revealed several DEGs associated with different types of cell death in the acute and subacute phases of injury, namely, in apoptosis pathways (*Mcl1*, *Ripk1*, *Casp8*, *Mapk8*, etc.), ferroptosis (*Acsl3*, *Slc39a14*, and *Slc3a2*), necroptosis (*Ripk1*, *Casp1*, *Casp8*, and *Mapk8*); and cellular senescence (*Map2k3*, *Akt3*, and *Mapk14*) was also evoked [159]. Additionally, using the scRNA database, the dynamics of interactions between immune and neuronal cells were analyzed, identifying key receptor–ligand interactions. Based on these data, an mRNA–miRNA–lncRNA regulatory network was constructed, and predictions of potential pharmacological interventions were made, including those already under investigation for SCI and SCI-associated neuropathic pain (e.g., N-acetyl-L-cysteine and capsaicin). The study demonstrates that various forms of cell death after SCI often co-exist and mutually influence each other, reinforcing the concept of PANoptosis in SCI (concurrent activation of multiple cell death pathways) and emphasizing the need for multi-targeted therapeutic strategies for an effective neuroprotective effect.

As another recent example of transcriptome being used to elucidate mechanisms of action of potential therapy, bulk RNA-seq was performed in a rat contusion SCI model after knockdown (KD) of *FL2* gene (potential therapy target, discussed above) [151]. Its downregulation via RNA interference led to improved locomotor function, and transcriptomic analysis revealed that *FL2* KD increases SCI-induced acute micro-glial response, supposedly leading to sharper inflammation resolution rather than resolution plateau, which is ultimately beneficial for functional improvement. Interestingly, *FL2* KD evoked significant changes in expression of *Car3*, *Cox2* (upregulation), and *Serhl2* (downregulation) genes. Apart from many other roles, Car3 is known to protect cells from hypoxia and oxidative stress [160]; in particular, cellular context can attenuate hypoxia-induced cell death via the PI3K/Akt/mTOR pathway [161]. Cox2 is mainly involved in prostaglandin synthesis during inflammation. Of note, perhaps scRNA-seq/snRNA-seq rather than bulk RNA-seq analysis would have revealed a more comprehensive picture of molecular events after Fl2 KD in SCI, as well as the combination of FL2 KD with targeting affected pathways, suggested by bulk RNA-seq.

However, there are also translational limitations of transcriptomics-nominated targets. For example, telmisartan was identified from a dorsal root ganglion conditioning-lesion regeneration signature, which may not fully reproduce spontaneous sensory neuron responses after clinically relevant SCI, because human dorsal root ganglion neurons show species-specific transcriptional organization and marker expression compared with rodents [162,163].

### 2.11. SCI Patient Transcriptome Profiling

A critical foundation for future human transcriptome profiling in SCI, an integrative part of a multiomics approach, will be the creation of multiple large-scale biobanks with comprehensive clinical data and biospecimen collections—including blood, cerebrospinal fluid, tissue samples, etc.—from SCI patients. The primary goal of such biobanks is to correlate detailed clinical data with molecular data and long-term patient outcomes to develop predictive models of injury severity and recovery, providing a foundation for future omics studies. For example, global RNA profiling from peripheral blood leukocytes was conducted in patients with acute SCI [164]. In this study, deep RNA-seq of white blood cells revealed that the transcriptomes of SCI patients diverge markedly from both healthy and non-CNS trauma controls. Differential expression analysis identified over 2000 genes that were significantly altered after SCI, of which 197 genes exhibited expression changes that correlated with clinical injury severity based on the American Spinal Injury Association (AIS) Impairment Scale. These severity-associated genes were enriched for immune response, cellular secretion, and localization processes; unsupervised co-expression network analysis revealed modules of coregulated genes whose combined expression patterns could predict severe (AIS A) or less severe (AIS D) injury with high accuracy. Notably, top candidate transcripts included immune-related genes such as *MMP8*, *HP*, and *KNG1*, highlighting systemic immune alterations in acute SCI. In a study by Li et al., 54 differentially expressed miRNAs (DEMs) and 1,656 DEGs in peripheral blood from SCI patients compared with healthy subjects were identified [165]. Functional enrichment analyses revealed that both DEMs and DEGs were associated with immune and inflammatory signaling pathways, including the neutrophil extracellular trap formation pathway, T-cell receptor signaling, and nuclear factor-κB (NF-κB) signaling. Three biomarker candidates, namely, *ANO10*, *BST1*, and *ZFP36L2*, whose expression levels changed significantly in the peripheral blood after SCI, demonstrated high discriminatory power in receiver operating characteristic (ROC) analysis.

Moreover, biobanking would facilitate recruitment of large-scale patient cohorts. This is why both national and international SCI-focused biobanks, including those collecting RNA to enable transcriptomic analysis, have been established to meet this demand [166].

In addition, a complementary approach to study human SCI pathobiology is development and use of in vitro 3D organoid SCI models derived from human-induced pluripotent stem cells (iPSCs) [167]. Particularly relevant models of different types of SCI (compressive contusion trauma, SC hemisection) can be based on recently established immune-competent 3D systems including erythro-myeloid progenitor cells (EMPs) [168]. Transcriptomics analysis was instrumental in the characterization of these 3D systems, determining the presence of several cell subpopulations within the organoid across a timeline, such as neurons, astrocytes, oligodendrocyte progenitor cells, and Schwann cells.

Overall, immune, inflammatory, and other gene expression patterns post-SCI and severity-associated transcriptional changes might highlight candidate pathways for intervention. This represents a necessary translational step toward rational target selection, treatment monitoring, and the eventual development of RNA- and pathway-directed therapies in future clinical trials. Moreover, transcriptomic and other omics data may also provide a key to understanding regenerative mechanisms, which, in turn, may lead to developing novel therapeutic agents.

### 2.12. Transcriptomics Combined with Other Omics Approaches

Over the past decade, high-throughput omics technologies beyond transcriptomics, namely, genomics, proteomics, metabolomics, epigenomics, etc., are increasingly being used in preclinical research, alone and in combination with transcriptomics. In the case of SCI, such an approach allows one to get a more robust and complex overview of the cell’s response to the injury/multitude of subsequent molecular events, revealing interconnections between genomics and transcriptomics, transcriptomics and proteomics, and so on and so forth.

For example, an integrative analysis of transcriptomic and metabolomic data enabled the identification of multi-element dysregulation of purine metabolism (ultimately affecting energy supply, oxidative stress, inflammation, etc.) in an acute SCI in a rat model [169]. Stemming from the observation that multiple metabolic failures occur after SCI, various therapeutic approaches based on the so-called metabolic reprogramming (or rather restoration) are being developed (as comprehensively reviewed in [168]).

A recent randomized clinical trial used a multiomics approach in the context of therapeutic rehabilitation [170]. Proteomic analysis of plasma and transcriptomic profiling of peripheral blood mononuclear cells (PBMCs) demonstrated that strength training modulates key immune pathways, including the complement system and humoral immune responses, in conjunction with improved motor function. Furthermore, subsequent mechanistic studies in a mouse model of exercise confirmed these findings and showed that plasma from exercised animals reduced demyelination and neuronal apoptosis after SCI, suggesting that systemic factors induced by exercise may exert a direct neuroprotective effect.

Further, a multiomic study revealed that AtN reprogramming via EE of transcription factors Ngn2, NeuroD1, and Ascl1 (a commonly used reprogramming approach) is impeded by transcription factor Olig2 [171], which is barely expressed by astrocytes and those expressions become elevated in cortical neurons after the aforementioned EE. Inhibiting Olig2 expression by RNAi resulted in a marked increase in NGN2-induced conversion of astrocytes to excitable neurons, which was demonstrated in astrocyte lineage-tracing mice. This finding has a direct translational application for CNS injury therapy based on cell reprogramming, proposing Olig2 as a target to improve AtN reprogramming efficiency.

Another potential target for SCI therapy, RPS3 (a 40S ribosomal subunit protein), was identified in a study combining RNA-seq and proteomic analysis which revealed the following mechanisms of liver-SC-NSCs crosstalk post-SCI: injury elevates levels of RPS3 in liver-derived EVs (LEVs), which inhibits NSCs differentiation via the NF-κB pathway [172]; it also drives activation of astrocytes, and knockdown of RPS3 in the liver promotes remyelination and axonal regeneration.

The study by Guo et al. demonstrated the key role of macrophage’s phenotypic transformation in SCI and defined its molecular mechanisms [173]. They demonstrated that metabolic crosstalk between endoplasmic reticulum stress (ERS) and mitochondria via the Ero1α–MAMs/Ca^2+^ axis promotes a persistent pro-inflammatory macrophage phenotype, including the release of mitochondrial DNA and activation of the cGAS–STING–NFκB cascade. Blocking this axis led to reduced inflammation and stimulated the transition of macrophages to M2 polarization. Thus, the integration of multiomics approaches enables the identification of key molecular mechanisms regulating immune-neuronal interactions after SCI.

Study by Song Y et al. demonstrated the important role of the pentose phosphate pathway (PPP) in maintaining homeostasis and regeneration of axons in peripheral sensory neurons [174]. The authors showed that PPP is active in the axoplasm of the sciatic nerve, where it ensures redox balance through NADPH production, supporting mechanosensory perception. After sciatic nerve injury, PPP participates in axon regeneration by providing ribose-5-phosphate for ribonucleotide synthesis. Unlike in the peripheral nervous system, PPP is not active after SCI, which contributes to the neuroregeneration failure. Reactivation of PPP via overexpression of transketolase in neurons or oral administration of ribose stimulated metabolic reprogramming, restored growth of sensory and motor axons, and improved neurological recovery after SCI.

Further, another study based on the analysis of both high-throughput transcriptomics and proteomics datasets proposed several potentially druggable therapeutic candidates for SCI; however, these findings await experimental validation [175].

Overall, until now, multiple studies of SCI biology have produced rich but rather disconnected omics-based insights. The key biological processes and molecular regulators in SCI (such as major signaling cascades, epitranscriptomics, proteomics, metabolomics, non-coding RNAs within a domain of transcriptomics, etc.) have traditionally been studied in isolation. At the same time, in other fields of biomedical research, Artificial Intelligence (AI) and Machine Learning (ML) are being increasingly used to integrate multiomics (and other) data, including transcriptomics. The use of AI in SCI studies is also becoming a research hotspot (comprehensively reviewed in [176]).

For example, ML analysis of the liquid chromatography-tandem mass spectrometry (LC–MS/MS) and transcriptomics data identified gene encoding Filamin A (a cytoskeleton-related protein involved in neural development) as a key regulator of neuronal apoptosis following SCI and therefore potential target for therapy [177].

**Figure 2:**
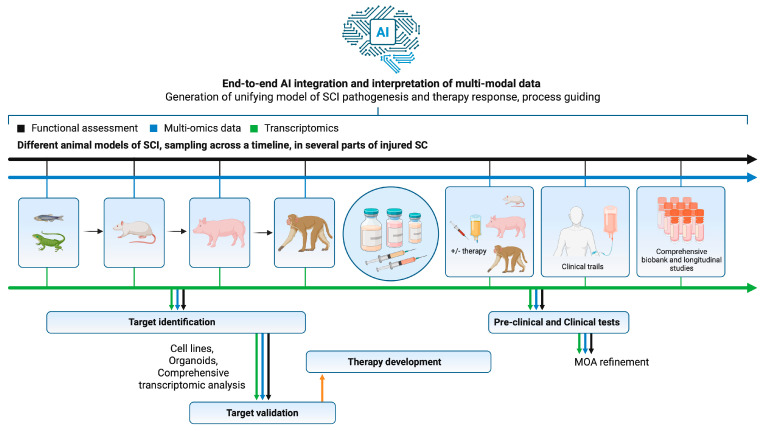
Proposed roadmap for SCI therapy development: from data integration to therapeutic targets. Created with BioRender.com. Retrieved from https://BioRender.com/dc2cgh9 (accessed on 19 June 2026). MOA—mechanism of action. The arrows represent the timeline.

We anticipate that the AI-driven approach will ultimately enable the discovery of complex biological relationships across multiple layers of the injury response in SCI and facilitate the development of unifying models that integrate regulatory mechanisms at the levels of RNA expression, protein dynamics, cell–cell interactions, signaling pathways, electrophysiology, etc. (Figure 2).

However, one of the major challenges hindering the application of AI in SCI research is the difficulty of gathering sufficient high-quality data [176]. Overcoming this challenge requires a coordinated and systemic approach. Perhaps future studies should aim at establishing large SCI-focused biobanks within collaborative research consortia, with rigorous standardization of sample collection protocols to ensure data integrity and consistency for AI training.

Nevertheless, we envision that integrating AI into SCI research is among the top priorities for the field over the next 5–10 years. Once again, a necessary prerequisite for this is generating large, high-quality multiomics datasets using standardized, reproducible methods across a variety of experimental animal models of SCI, as well as from patient-derived samples, including those from longitudinal, preclinical, and clinical studies evaluating different therapies, within corresponding research consortia. Here, to avoid redundancy, we refer the readers to recent comprehensive reviews on integrative omics-based approaches to SCI, including AI-driven therapeutics ([178] and others).

## 3. Conclusions

It is evident that transcriptome data (especially when combined with other omics data) are instrumental to identify key genes, regulatory biomolecules, pathways, and cell subpopulations involved in the pathophysiology of SCI and post-injury neuroregeneration. It can also define mechanisms of action of novel therapies (including cell-based therapies) and guide therapy strategies, especially when using “targeted” approaches such as direct in situ reprogramming.

To summarize, the potential of transcriptomics insights into SCI for therapy development is illustrated by several examples presented in (Table 1).

Apparently, our current understanding of the field is incomplete. Some domains of transcriptomics are understudied (for example, the role of rRNA or other less studied ncRNAs), there is no unifying model integrating multiomics data. SCI-associated systemic syndromes remain understudied by transcriptomics methods, despite being among the major determinants of long-term health, independence, and quality of life for people living with SCI. These complications are common, clinically consequential, and incompletely managed by current therapies. This dictates a need and creates an opportunity for future transcriptomic research in the field of SCI.

Despite all the recent advances in molecular and computational approaches to transcriptomics, and a wealth of animal models of SCI, there are also translational limitations of transcriptomics-nominated targets and of currently used animal models of trauma, as well as translational failures. They likely result from discrepancies between currently used animal models and human pathophysiology, predominant use of bulk RNA-seq masking cell-type-specific responses and dynamic changes in heterogeneous tissues, limited integration of multiomics data, and, finally, lack of validation across diverse datasets/protocol standardization, leading to poor reproducibility. In our opinion, this should be addressed by integrating multiomics approaches and by developing more clinically relevant models. Additionally, using AI for large dataset integration, target identification, and validation could significantly improve and facilitate the clinical translation of findings. The creation of large biobanks within SCI-research consortia will enable standardizing sample collection protocols and facilitate data sharing, ultimately resulting in generation and comprehensive analysis of robust, large-scale high-quality datasets. Crucially, such SCI-focused research consortia can bridge resource gaps by enabling strategic collaborations, connecting investigators with access to unique clinical or preclinical SCI samples, as well as various in vitro and in vivo models, with all those possessing specialized equipment or mastered advanced transcriptomic techniques. Such synergy could ensure that various biological materials are analyzed with a range of state-of-the-art complementary methods, maximizing research output.

## Figures and Tables

**Table 1:** Transcriptomics-driven therapeutic targets and interventions in SCI.

Target	Role in SCI	Proposed Therapeutic Strategy	Proposed or Tested Therapeutic Intervention	Transcriptomic/ Epitranscriptomic Method	Validation of Therapeutic Strategy/Intervention	References
Cell death/survival/proliferation						
CDK1	Cell-cycle reactivation post-injury; neuronal apoptosis	Inhibition	Olomoucine; Flavopiridol	Meta-analysis of several RNA-seq studies	Previously shown to reduce glial proliferation and secondary neuronal loss	[54]
p21	Limits NSPC cells proliferation	Gene knockdown	RNAi (p21)	scRNA-seq	Increased NSPC proliferation and migration, tissue cable formation in T9 SCI in rats, accelerated locomotor recovery	[127]
Regeneration (axonal, CST) and neurogenesis						
G3BP1	Suppresses axonal mRNA translation via stress granule sequestration	Functional Modulation	Disrupting G3BP1 function with a dominant-negative approach	RNA immunoprecipitation, ddPCR	Increased axon growth in cultured neurons, accelerated nerve regeneration in vivo	[68]
Olig2	Limits astrocyte-to-neuron (AtN) conversion; knockdown enables AtN reprogramming	Gene knockdown	RNAi (Olig2)	scRNA-seq	Increased AtN conversion ~3-fold	[171]
FL2	Negative regulator of axon regeneration	Gene knockdown	siRNA-FL2 nanoparticles	Bulk RNA-seq	Modest locomotor recovery with preserved corticospinal function, reduced inflammation, more oligodendrocytes	[151]
Repulsive Guidance Molecule BMP Co-Receptor A signaling	Inhibits axon growth after SCI	BMSC-derived exosomes	CD271^+^CD56^+^ BMSC-derived exosomes in hydrogel	MicroRNA array assay of EV cargo, scRNA-seq, miRNA in situ hybridization	Promoted axonal regeneration after SCI in vivo	[115]
Complex inhibition of axon regeneration program in DRG neurons	Inhibits axonal regeneration	Drug repurposing, downregulation of AGTR1; AT1R antagonists	Telmisartan	Gene expression microarrays	Promoted axonal regeneration after SCI in vivo, improved the sensory and motor function recovery in mice with SCI	[149]
METTL14- mediated Trib2 m6A RNA modification	Regulates expression of Trib2, involved in MAPK signaling	METTL14 stabilization	Syringin	m^6^A RNA Methylation Quantification, meRIP-qPCR	Enhanced CST regeneration, promoted neurological recovery	[109]
CircRNA_01477	Upregulated in SCI; modulates axonal growth via the miRNA-3075/FosB	Knockdown	CircRNA_01477 knockdown, FosB knockdown	FISH, RNA-seq	Significantly increased length of axons	[94]
Glia functions, Neuroinflammation						
OPN–CD74 axis	Tregs modulated microglial phagocytic function/synaptic engulfment post-SCI.	Axis inhibition, blockade of OPN–CD74 interaction	Genetic ablation of Cd74	scRNA-seq	Enhanced synaptic engulfment, improved neurological outcomes post-SCI	[155]
CD44, NCL	Neuroinflammation; astrocyte activation	Inhibition	CD44 and NCL as targets	scRNA-seq, snRNA-seq	Attenuation of astrocyte reactivity and proliferation in vivo and in vitro by NCL inhibitor GroA/AS1411 aptamer	[156], functional validation in [157]
B2m, Itgb5, Vav1	Immune hub genes in microglia/ macrophages	Modulation of function of B2m, Itgb5, Vav1 proteins	Low-dose Decitabine	bulk-RNA-seq, scRNa-seq	Decitabine treatment in mice after SCI reduced levels of pro-inflammatory factors, increased anti-inflammatory factors, induced M1-to-M2 polarization of macrophages/microglia, improved neurological function and electromyography	[150]
EV Transcriptome/EV-mediated signaling						
WNT10b signaling mediated by miR-152-3p	Cognition and neurogenesis modulated by microglia EV signaling disrupting neurogenesis in dentate gyrus	Modulation of WNT pathway	WNT agonist	miRNA-seq	Promoted neurogenesis and improved cognitive functions in mice with SCI	[114]

## Data Availability

No new data were created or analyzed in this study. Data sharing is not applicable to this article.

## Abbreviations

ac4C	N4-acetylcytosine
AD	autonomic dysreflexia
AIS	American Impairment Scale
AtN	astrocyte-to-neuron conversion
AtiN	astrocyte-to-induced neuron conversion
BDNF	brain-derived neurotrophic factor
BSI	brain–spine interface
cAMP	cyclic adenosine monophosphate
Cc	central channel
CDK1	cyclin-dependent kinase 1
circRNAs	circular RNAs
CNS	central nervous system
CRISPR-TO	clustered regularly interspaced short palindromic repeats transcriptome organization
CSPG	chondroitin sulfate proteoglycan
CST	corticospinal tract
ddPCR	digital droplet PCR
DEGs	differentially expressed genes
DEMs	differentially expressed miRNAs
DERs	differentially expressed RNAs
DMAPT	dimethylamino parthenolide
DRG	dorsal root ganglion
EE	ectopic expression
Evs	extracellular vesicles
EMPs	erythro-myeloid progenitor cells
ExSeq	expansion sequencing
ERS	endoplasmic reticulum stress
FISH	fluorescence in situ hybridization
GJCs	gap junctional channels
GRO-seq	global run-on sequencing
hUC-MSCs	human umbilical cord-derived mesenchymal stromal/stem cells
iN	induced neuron
iPSCs	induced pluripotent stem cells
KD	Knockdown
LAR	leukocyte antigen-related
LEVs	liver-derived extracellular vesicles
lncRNAs	long non-coding RNAs
LC–MS/MS	liquid chromatography-tandem mass spectrometry
m6A	N6-methyladenosyne
m6Am	N6,2′-O-dimethyladenosine
m5C	5-methylcytosine
m7G	N7-methylguanosine
MAPK	mitogen-activated protein kinase
miRNAs	microRNAs
MCEMP1	mast cell-expressed membrane protein 1
METTL14	methyltransferase-like 14
METTL16	methyltransferase-like protein 16
MOA	mechanism of action
MSE	microtubule severing enzyme
MSCs	mesenchymal stromal/stem cells
NBD	neurogenic bowel dysfunction
ncRNA	non-coding RNA
NCL	nucleolin
NF-κB	nuclear factor-κB
NMN	nicotinamide mononucleotide
NNT	neuroactive network tissue
NRV	non-regenerative vertebrates
NSCs	neural stem cells
NSPCs	neural stem progenitor cells
PBMCs	peripheral blood mononuclear cells
PBM	Photobiomodulation
PI3K	phosphoinositide 3-kinase
piRNAs	Piwi-interacting RNAs
PRO-seq	precision nuclear run-on sequencing
PSCSR-seq	parallel single-cell small RNA-sequencing
PPP	pentose phosphate pathway
RBPs	RNA-binding proteins
RIP-seq	RNA-immunoprecipitation sequencing
RNAi	RNA interference
ROC	receiver operating characteristic
RTP	regenerative transcriptome profile
RV	regenerative vertebrates
SC	spinal cord
SCI	spinal cord injury
SCI-IDS	spinal cord injury-induced immunodeficiency syndrome
scRNA-seq	single-cell RNA-sequencing
snRNA-seq	single-nucleus RNA-sequencing
snoRNAs	small nucleolar RNAs
snRNAs	small nuclear RNAs
sRNA-sequencing	small non-coding RNA-sequencing
tsRNAs	transfer RNA-derived small RNAs
TRACK-SCI	Transforming Research and Clinical Knowledge in Spinal Cord Injury
TRAP	translating ribosome affinity purification
TNTs	tunneling nanotubes
OPN	Osteopontin
